# Fasciite nécrosante de la nuque, une forme clinique rare des cellulites cervico-faciales : à propos d'un cas au Togo

**DOI:** 10.48327/mtsi.v3i2.2023.303

**Published:** 2023-04-18

**Authors:** Haréfétéguéna BISSA, Essobiziou AMANA, Koffi Dzidzo Jude AMEGBLE, Hervey REOULEMBAYE DJIM, Winga FOMA

**Affiliations:** 1 Service de chirurgie maxillo-faciale, Centre hospitalier universitaire d'Aneho, Togo; 2 Service d'oto-rhino-laryngologie et chirurgie maxillo-faciale, Centre hospitalier universitaire Sylvanus Olympio de Lomé, Togo; 3 Service des brûlés, des plaies et de la cicatrisation, Centre hospitalier universitaire Sylvanus Olympio de Lomé, Togo

**Keywords:** Nuque, Cellulite, Fasciite nécrosante, Greffe, Lambeau, Togo, Afrique subsaharienne, Neck, Cellulitis, Necrotizing fasciitis, Graft, Flap, Togo, Sub-Saharan Africa

## Abstract

Nous rapportons le cas d'un patient de 75 ans, diabétique, ayant présenté une fasciite nécrosante cervicale postérieure compliquant une cellulite. Une prise en charge médicale en soins intensifs et un drainage chirurgical ont été entrepris; une excision séquentielle des tissus nécrotiques a laissé une grande perte de substance de la région nucale pour laquelle nous avons opté pour une cicatrisation dirigée dans un premier temps. La couverture définitive de cette perte de substance par lambeau locorégional de rotation ou par greffe de peau mince est discutée. Cependant, elle a été refusée par le patient.

## Abstract (en)

We report the case of a 75-year-old diabetic patient who presented with posterior cervical necrotizing fasciitis complicating cellulitis. Medical management in intensive care and surgical drainage were undertaken; sequential excision of the necrotic tissue left a large loss of substance of the nuchal region for which we opted for directed healing in the first instance. The definitive coverage of this loss of substance by locoregional rotation flap or by thin skin grafting was discussed. However, it was refused by the patient.

## Introduction

Les cellulites cervico-faciales sont des affections inflammatoires polymicrobiennes, développées dans le tissu cellulo-adipeux de la face et du cou [4]. En l'absence de traitement, elles peuvent évoluer vers une nécrose extensive des fascias et des tissus mous constituant ainsi une véritable fasciite nécrosante. Cette pathologie d'apparence banale au début peut engager le pronostic vital par son évolution fulminante. Elle constitue une réelle urgence médico-chirurgicale [1] dont le pronostic dépend de plusieurs facteurs, notamment le terrain, la rapidité et la qualité de la prise en charge initiale adaptée au stade évolutif [4]. Les localisations préférentielles sont la face et la région antérieure du cou. La localisation nucale isolée est rare. Nous rapportons un cas de fasciite nécrosante isolée de la nuque chez un patient de 75 ans.

## Cas Clinique

**Figure 1:**
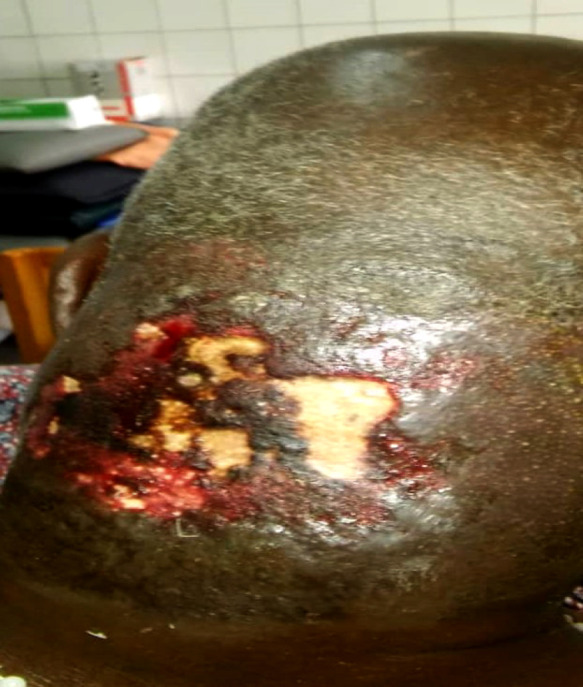
Cellulite nucale suppurée Suppurative nuchal cellulitis

**Figure 2:**
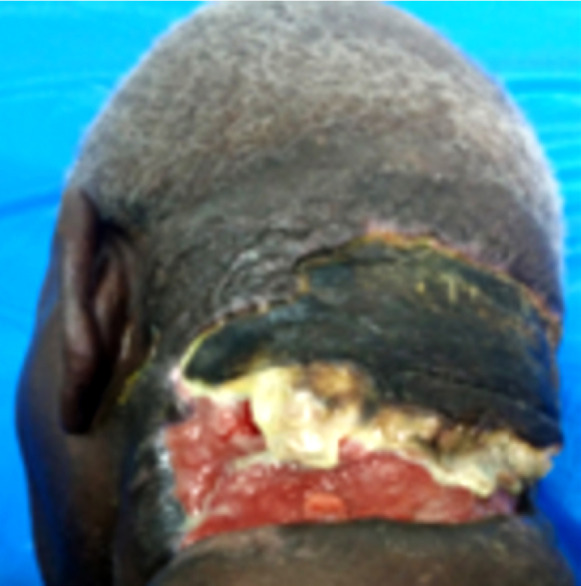
Fasciite nécrosante nucale Posterior cervical necrotizing fasciitis

**Figure 3:**
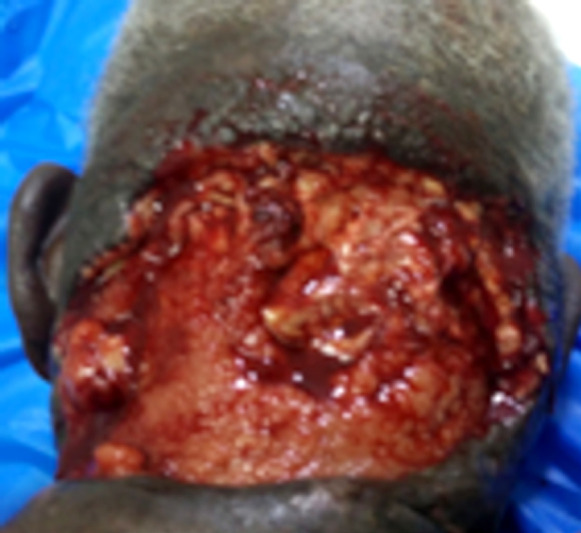
Perte de substance (après excision de tissus nécrotiques) Loss of substance (after excision of necrotic tissue)

**Figure 4:**
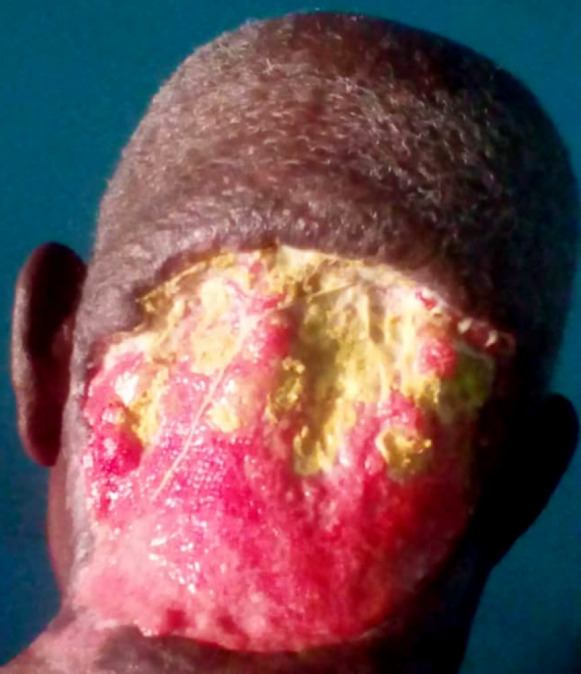
Plaie après 1 mois de cicatrisation dirigée Wound after 1 month of directed healing

Il s'agissait d'un patient de 75 ans, admis au CHU Sylvanus Olympio de Lomé (Togo) pour altération de l’état général et de la conscience précédée par un torticolis dans un contexte fébrile évoluant 6 jours avant l'admission. Il n'avait pas d'antécédents pathologiques connus. Des infusions à base d'herbe ont été appliquées à la région cervicale. Le patient a été admis en réanimation. L'examen clinique a permis de noter une fièvre à 39 ^o^C, une asthénie avec une anorexie importante, un trouble de conscience avec un score de Glasgow évalué à 12/15, une raideur de la nuque sans signes de Kernig ni de Brudzinski. L'examen de la région cervicale a permis de noter une tuméfaction douloureuse et fluctuante de la région nucale avec des zones de dermabrasions (Fig. 1). La cavité buccale et l'oropharynx étaient normaux ainsi que l'examen des autres appareils. La ponction exploratrice a ramené un pus qui a été envoyé pour examen cytobactériologique. La glycémie à jeun était de 3 g/dl, la numération formule sanguine a noté une hyperleucocytose à polynucléaires neutrophiles à 22 500 par mm^3^, le bilan rénal et hépatique, l'ionogramme sanguin, la sérologie rétrovirale ainsi que la radiographie thoracique étaient normaux. Devant ce tableau, le diagnostic de cellulite nucale isolée sur terrain de déséquilibre glycémique a été retenu. Un drainage chirurgical sous anesthésie locale a été réalisé avec lavage abondant à l'eau oxygénée et au Dakin. Une antibiothérapie probabiliste première à base de ceftriaxone 2 g par 24 h et métronidazole 500 mg matin et soir a été instituée puis réadaptée après antibiogramme qui a isolé le *Staphylococcus aureus* sensible aux macrolides (azithromycine 500 mg matin et soir pendant 10 jours). L’évolution après 2 semaines d'hospitalisation a été marquée par l'amélioration de l’état général et de la conscience avec stabilisation de la glycémie sous insulinothérapie. L'examen clinique de la région cervicale postérieure a noté une nécrose de la peau nucale étendue au scalp occipital (Fig. 2). Le diagnostic de fasciite nécrosante isolée de la nuque sur terrain de déséquilibre glycémique compliquant une cellulite a été retenu. Nous avons effectué un débridement au bloc opératoire. L'excision réalisée a permis de noter une perte de substance cutanée ovalaire évaluée à 7 cm de rayon soit 154 cm^2^ de surface, propre, sans exposition des organes nobles (Fig. 3). Nous avons opté pour une cicatrisation dirigée (Fig. 4 et 5), suivie d'une greffe de peau mince pour la couverture. Cependant, le patient a renoncé à cette prise en charge chirurgicale.

**Figure 5:**
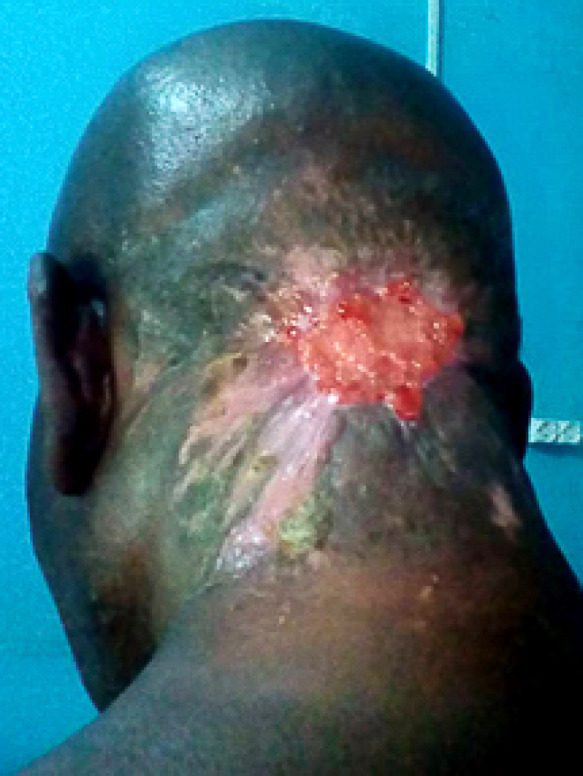
Plaie après 4 mois de cicatrisation dirigée (cicatrice rétractile) Wound after 4 month of directed healing (retractile scar)

## Discussion

Les cellulites cervico-faciales représentent 16 % des consultations et la première cause d'hospitalisation et de décès dans notre service ORL et chirurgie cervico-maxillo-faciale du CHU Sylvanus Olympio de Lomé (Togo). La région péri-mandibulaire est la localisation la plus fréquente dans notre pratique ainsi que dans les études portant sur les cellulites cervico-faciales [2]. La localisation nucale a été rarement retrouvée, ce qui fait la particularité de notre observation. Le diabète est un facteur acquis favorisant la survenue des cellulites et par extension des fasciites nécrosantes. Il est le facteur favorisant dans notre observation.

Le torticolis fébrile chez l'adulte est plus rare que chez l'enfant. Très rarement, ce signe fonctionnel fait appel à une cellulite nucale isolée.

Le traitement de la cellulite cervico-faciale suppurée est médico-chirurgical. L'anti-biothérapie est fonction du germe. Quant au traitement chirurgical, il consiste en un drainage chirurgical avec discision à la pince hémostatique ou au doigt des tissus sous-cutanés permettant l'effondrement des cloisonnements, et enfin mise en place d'un système de drainage. Dans la région nucale, l'importance du tissu cellulo-adipeux et du système musculaire impose le suivi régulier des étapes sus-citées avec des pansements réguliers afin de préserver le pronostic vital. Dans le cas des fasciites nécrosantes avec perte de substance cutanée de grande surface, un lambeau ou une greffe de peau est recommandée [3, 5].

## Contributions Des Auteurs

Tous les auteurs ont contribué à ce travail et ont lu et approuvé la version finale du manuscrit.

## Liens D'intérêts

Les auteurs ne déclarent aucun conflit d'intérêts.
